# Coronary heart disease mortality is decreasing in Argentina, and Colombia, but keeps increasing in Mexico: a time trend study

**DOI:** 10.1186/s12889-020-8297-5

**Published:** 2020-02-03

**Authors:** C. Arroyo-Quiroz, T. Barrientos-Gutierrez, M. O’Flaherty, M. Guzman-Castillo, L. Palacio-Mejia, E. Osorio-Saldarriaga, A. Y. Rodriguez-Rodriguez

**Affiliations:** 1 Center for Population Health Research, National Institute of Public Health, Av Universidad 655 Col. Sta Ma Ahuacatitlán, 62100 Cuernavaca, Mor, CP Mexico; 2 Universidad Autonoma Metropolitana- Unidad Lerma, Lerma de Villada, Mexico; 3 Institute of Psychology, Health and Society, University of Liverpool, Liverpool, UK; 4 CONACYT- National Institute of Public Health, Cuernavaca, Mexico; 5 Ministry of Health, Bogota, Colombia

**Keywords:** Coronary disease mortality, Statistics and numerical data, Latin America, Trends

## Abstract

**Background:**

Mortality rates due to coronary heart disease (CHD) have decreased in most countries, but increased in low and middle-income countries. Few studies have analyzed the trends of coronary heart disease mortality in Latin America, specifically the trends in young-adults and the effect of correcting these comparisons for nonspecific causes of death (garbage codes). The objective of this study was to describe and compare standardized, age-specific, and garbage-code corrected mortality trends for coronary heart disease from 1985 to 2015 in Argentina, Colombia, and Mexico.

**Methods:**

Deaths from coronary heart disease were grouped by country, year of registration, sex, and 10-year age bands to calculate age-adjusted and age and sex-specific rates for adults aged ≥25. We corrected for garbage-codes using the methodology proposed by the Global Burden of Disease. Finally, we fitted Joinpoint regression models.

**Results:**

In 1985, age-standardized mortality rates per 100,000 population were 136.6 in Argentina, 160.6 in Colombia, and 87.51 in Mexico; by 2015 rates decreased 51% in Argentina and 6.5% in Colombia, yet increased by 61% in Mexico, where an upward trend in mortality was observed in young adults. Garbage-code corrections produced increases in mortality rates, particularly in Argentina with approximately 80 additional deaths per 100,000, 14 in Colombia and 13 in Mexico.

**Conclusions:**

Latin American countries are at different stages of the cardiovascular disease epidemic. Garbage code correction produce large changes in the mortality rates in Argentina, yet smaller in Mexico and Colombia, suggesting garbage code corrections may be needed for specific countries. While coronary heart disease (CHD) mortality is falling in Argentina, modest falls in Colombia and substantial increases in Mexico highlight the need for the region to propose and implement population-wide prevention policies.

## Background

Coronary heart disease (CHD) has been the leading cause of death worldwide since 1970, accounting for 7.3 million deaths in 2010 and 13% of global mortality [1]. The current distribution of CHD responds to a large reduction in CHD deaths in high-income countries, and mixed trends in low and middle income countries (LMIC) [1, 2]. In most European countries a 30% decline in CHD deaths was registered from 1980 to 2010; similar reductions were observed in the US, Canada, and Australia, where CHD rates are now less than half of 1980s rates [3, 4]. In contrast, increasing trends were observed in Eastern Europe, Asian and Latin-American countries [4–7]. Differences in trends have been explained by changes in the prevalence of risk factors and differential access to treatments [3, 5, 8–12].

The behavior of the CHD epidemic not only differs across countries, but across age groups. A recent discussion has arisen about the possibility that CHD mortality in young adults could be stabilizing or even increasing. While in the US, UK and Australia, CHD mortality in young adults (< 55 years) seems to be decreasing or remaining constant [3, 10, 13], this behavior is not common to all high income countries, and some of them are actually experiencing increases [14]. In the case of LMIC, little is known about mortality in young adults. In a recent study in Latin America on adults aged 35–44 years, most countries showed declining trends, except for Mexico and Panama [7]. In this study, the highest decreases in CHD mortality rates in men between 2001 and 2013 were in Ecuador (46% from 12.6 to 6.8 per 100,000), Puerto Rico (32% from 16.7 to 11.3 per 100,000) and Argentina (28% from 14.8 to 10.6 per 100,000) [7]. In the case of women, the highest decreases were in Ecuador (59% from 5.2 to 2.1 per 100,000), Uruguay (51% from 4.3 to 2.1 per 100,000), Colombia (35% from 7.1 to 4.7 per 100,000) and Cuba (35% from 7.4 to 4.8 per 100,000) [7]. In contrast, Panama showed the highest increases in both sexes (42% in men and 52% in women) followed by Mexico (18% in men and 4% in women) [7]. Although the changes in Panama were higher, the CHD mortality rates are still lower in Panama than in Mexico for both sexes in this age-group [7].

Latin America shows complex patterns for CHD mortality. Countries are at different stages of the CHD epidemic, and while most of them show declines some others are experiencing increases [4, 7, 15]. Some studies have analyzed age- and sex-specific mortality rates [7, 15, 16]; however, they have studied age-groups by life stages, which are not as detailed as decennial age groups. Also, studies tend to exclude adults younger than 35 years old. Understanding how mortality trends evolve over a long period is crucial to further understand potential drivers of the epidemic at a national level. Importantly, previous studies looking at mortality trends did not consider the differential quality of death registries across Latin American countries, an aspect that could account for some of the observed trend differences.

We aimed to examine and compare age-adjusted, age- and sex-specific, and garbage code corrected mortality trends for CHD from 1985 to 2015 in three Latin American countries: Argentina, Colombia, and Mexico. These countries are in the top-five gross domestic product in the region and have comparable health investment [17–19]. They also have marked differences in data quality and CHD mortality levels in 1985: Mexico with low, Argentina intermediate and Colombia at high mortality rates. We analyzed the mortality rates of these countries to contrast age- and sex-specific differences, specifically to evaluate the hypothesis that CHD mortality has stagnated in young adults. Finally, we aimed to analyze the influence of garbage-code correction in this comparison.

## Methods

Demographic and mortality data were obtained from the World Health Organization (WHO) database [20]. The underlying cause of death from coronary heart disease was determined using the International Classification of Diseases (ICD), codes 410–414 from the 9th and codes I20-I25 from the 10th revision, all codes were pooled together in a category of “all coronary heart diseases”. Data for all CHD deaths (1985–2015) in the 3 countries were grouped by year, sex and age (decennial group) for adults aged 25 years and over.

Age and sex-specific mortality rates were calculated as the number of deaths in each age-sex group by the total number of persons in the population group per 100,000 inhabitants per year and country. Age standardization of rates was achieved through the direct method, multiplying the age-specific mortality rates by the proportion of people in each age group in the world standard population [21]. This methodology was repeated within each sex to obtain age standardized rates by sex.

Standardized CHD mortality rates and age and sex specific mortality rates for each country were analyzed using joinpoint regression to identify years at which changes in mortality trends occurred [22, 23]. Joinpoint regression is a statistical method used to identify years at which changes in mortality trends occurred. The estimated annual percentage change (APC) was computed by fitting a regression line to the natural logarithm of the rates and using the calendar year as an independent variable*.* A 95% confidence interval for the APC was also computed. We used a Bayesian Information Criterion (BIC) approach to select the model that best fitted the data. The analysis was performed using software developed by the Surveillance Research Program of the US National Cancer Institute (Joinpoint version 3.4.1) [24].

We performed an analysis to quantify how garbage-codes influenced mortality rates. The term “garbage- codes” (GC) has been used for mortality codes that correspond to causes that are not useful for the analysis of deaths in public health, since they are ambiguous or nonspecific, or refer to symptoms or intermediate causes [25, 26]. Methods to redistribute GC have been proposed. Previous studies have shown that CHD mortality rates are particularly prone to changes after redistribution [27, 28].

Garbage-code information was determined using codes 428, 428.0, 428.1 and 428.9 for ICD 9 and codes I50, I50.0 I50.1 I50.9 for ICD 10 (Appendix). All codes were pooled together into one category (GCMort), data in every country were grouped by year, sex and age (10-year groups) for adults aged ≥25 years. Argentina and Colombia had information from 1997 to 2015, so we restricted our analysis to this period. Garbage-code corrected mortality was estimated according to the Global Burden of Disease (GBD) methodology [27, 28] in which:

$$ {\mathrm{CHDMort}}_{\mathrm{Corrected}}={\mathrm{CHDMort}}_{\mathrm{O}}+\updelta \mathrm{GCMort} $$

Where.

*CHDMort*_*Corrected*_ is CHD mortality corrected by garbage codes;

*CHDMort*_*0*_ is the original CHD mortality;

*δ* is the redistribution percentage of garbage code by age and sex group according to GBD methodology and;

*GCMort* is the garbage code mortality related to CHD.

The redistribution percentage (δ) for the age group from 15 to 49 years in developing countries was 72 and 47% for men and women respectively, for the group over 50 the percentage was 74% for men and women [28]. Standardized mortality rates and age and sex specific garbage-code corrected mortality rates for each country, were obtained and analyzed using joinpoint regression in order to compare them with the uncorrected rates. Finally, we compared the garbage-code corrected and the original CHD mortality rates for every country, sex and age-group using the Kruskal-Wallis non-parametric test.

### Ethical consideration

Our study is a secondary analysis of existing data from the World Health Organization (WHO) mortality database. The datasets used for this study are publicly available and anonymous.

## Results

**Fig. 1:**
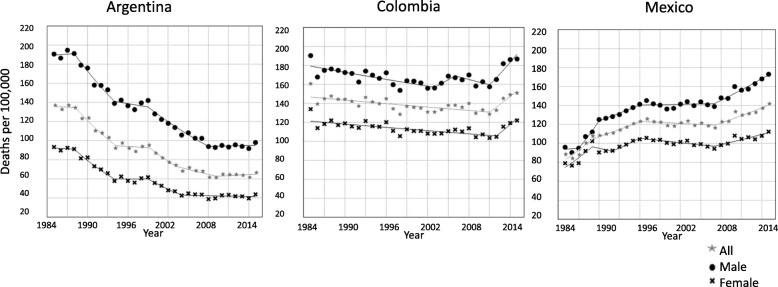
Trends in age-standardized mortality rates per 100,000 by sex for coronary heart disease. Argentina, Colombia, and México. 1985–2015. *Star*: all; *circle:* male; *cross:* female

In 1985, mortality rates were higher in Colombia (160.6 per 100,000) and Argentina (137.6 per 100,000) and lower in Mexico (86.5 per 100,000). Between 1985 and 2015 age-adjusted CHD mortality rates declined in Colombia (6.5%) and Argentina (51.1%) but increased in Mexico by 60.8% (Fig. 1). By 2015, mortality rates were highest in Colombia (150.7 per 100,000), followed by Mexico (140.8 per 100,000) and Argentina (67.3 per 100,000). The steepest decline was observed in Argentinian women and the sharpest increase in Mexican men. In all countries, men had higher mortality rates than women during the study period; in Colombia, the difference between men and women remained parallel and unchanged, in Argentina the gap between men and women decreased, while in Mexico the initial gap between men and women kept increasing.

The decline in CHD mortality rates was more pronounced in Argentina, where women had stronger reductions than men (53% vs 48.6%). Four joinpoints were obtained in the analysis by sex (Table 1 in Appendix). The largest reductions were observed between 1985 and 1994 for both men (APC -4.9; 95%CI -6.5, − 3.4) and women (APC -6.7; 95%CI -9.3, − 4.1). From 2008 to 2015, CHD mortality in Argentina seems to have stabilized (APC 0.2; 95%CI -0.9, 1.2).

In Colombia, a small reduction in CHD mortality was observed on average over the 1985–2015 period, for both men (1.9%) and women (9.8%). In women, at the beginning of the period, from 1985 to 2012, the APC was negative (APC -2.5; 95%CI -3.3, − 0.7), but became positive in the 2012 to 2014 period (APC 1.1; 95%CI -1.6, − 0.6). In men, we observed an initial negative trend from 1985 to 2003 (APC -0.7; 95%CI -1.1, − 0.4), a stabilization period from 2003 to 2011 with little change, followed by an increasing trend from 2011 to 2015 (APC 4.7; 95%CI 2.3, 7.2).

From 1985 to 2015, the global increase over the study period in Mexico was 80.6% in men and 41.9% in women. In the joinpoint analysis, five changes in trend were obtained for both men and women. The highest increase in CHD mortality in men was observed between 1987 and 1990 (APC 9.3; 95%CI 0.4, 19), while for women it was from 1985 to 1989 (APC 6.6; 95%CI 2.6, 10.7). From 1996 to 2007 mortality stabilized in men, while a significant decline was observed in women (APC -0.8; 95%CI -1.4, − 0.1). From 2007 to 2015 men experienced a small significant increase (APC 2.5; 95%CI 1.9, 3). Women had a significant increase from 2007 to 2012 (APC 1.8; 95%CI 1, 2.5).

**Fig. 2:**
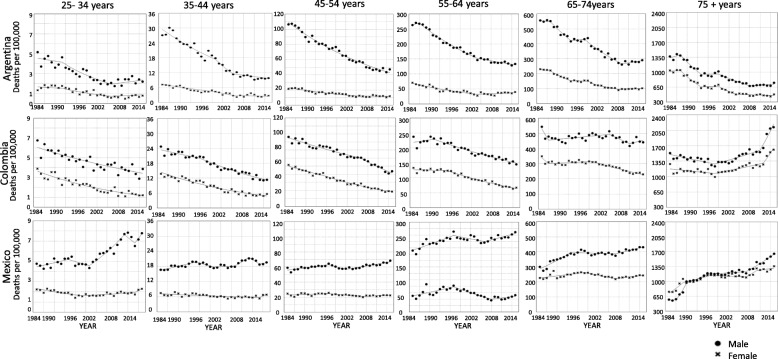
Trends in age and sex-specific mortality rates per 100,000 for Coronary Heart Disease, by country. Argentina, Colombia, and México. 1985–2015. *Circle:* male; *cross:* female

In Argentina and Colombia age- and sex-specific mortality trends were similar across strata (Table 2 in Appendix). However, two groups showed important discrepancies (Fig. 2): (i) Argentinian men and women aged 25–34 had a non- significant increase in mortality at the end of the period studied (APC 1.8; 95%CI − 1.9, 5.6 and APC 1.9; 95%CI 4, 8.1 respectively); and (ii) Colombians aged 75 and more had an initial decrease in rates from 1985 to 1988, followed by a sharp increase (men from 2011 to 2015 APC 8.3; 95%CI 5.5, 11.3; women from 2012 to 2015 APC 8.1; 95%CI 4, 12.4).

In the case of Mexico, some age- and sex-specific groups differed from the average trends. Mexican men aged 25–34 experienced an increase in mortality rates during most of the study period; the highest increase was observed from 2000 to 2010 (APC 5.2; 95%CI 3.8, 6.7), there was also a positive trend from 2013 to 2015, but it was non-significant (APC 6.5; 95%CI -5.7, 20.3). Mortality rates in young Mexican women aged 25–34 had a small significant increase between 1998 and 2013 (APC 1.2; 95%CI 0.3, 2.2). In men and women aged 35 and older, mortality rates kept increasing during the study period, particularly in those aged 75 and older, the largest increases in these age groups were observed between 1987 and 1990 in men (APC 19.3; 95%CI 6.1, 34.2) and 1987–1989 in women (APC 7.6; 95%CI 2.7, 12.8).

**Fig. 3:**
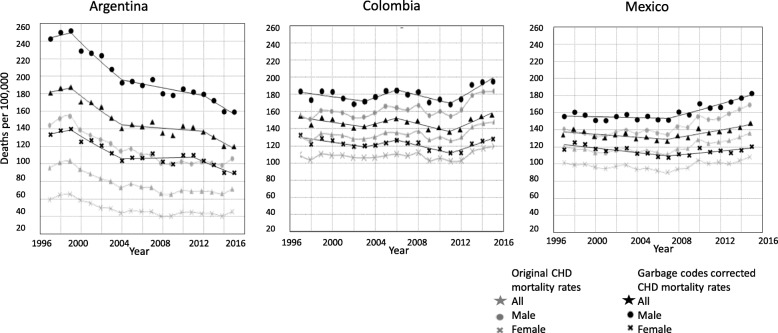
Trends in adjusted age-standardized mortality rates per 100,000 by sex for coronary heart disease corrected by garbage codes. Argentina, Colombia, and México. 1997–2015. *Gray:* original mortality rates; *black:* garbage-codes corrected mortality rates. *Star*: all; *circle:* male; *cross:* female

**Fig. 4:**
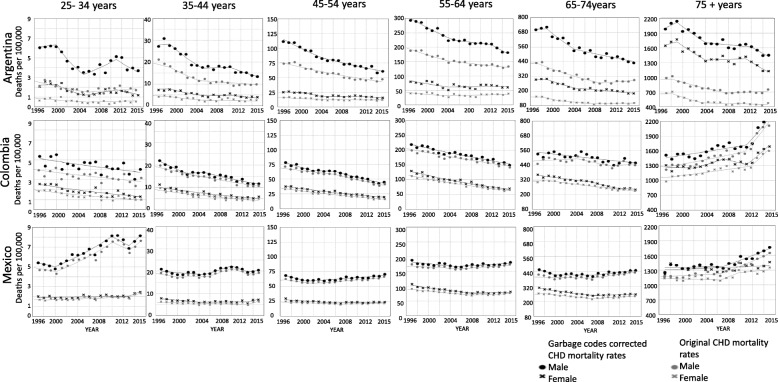
Trends in age and sex-specific mortality rates per 100,000 for Coronary Heart Disease corrected by garbage codes, by country. Argentina, Colombia, and México. 1997–2015. *Gray:* original mortality rates; *black:* garbage-codes corrected mortality rates. *Circle:* male; *cross:* female

Garbage-code correction had an important influence on the intercepts of the trends but little effect on the slopes (see Fig. 3). In Argentina, garbage-code correction produced an increase in the number of cases across the study period: on average, per year there were approximately 77 additional deaths per 100,000, compared with 12.5 additional deaths in Colombia and 12.8 in Mexico. The differences between the corrected and the original mortality rates were significant in the case of Argentinian men and women; in contrast, the differences in Colombia and Mexico were not significant (Table 2 in Appendix). Relative to the original rates, garbage-code correction implied an average increase in rates of 104.6% in Argentina, 9.4% in Colombia and 10.2% in Mexico. In Argentina, garbage-code corrections increased mortality rates in men by 83.8%, compared to 140.4% in women (Table 2 in Appendix); these large differences were not observed in Colombia (men 8.5%, women 10.6%) or Mexico (men 8.2%, women 12.8%). While studying age and sex-specific mortality rates (Fig. 4), in the three countries the increases due to the correction were higher in the youngest and oldest age-groups. Women in the three countries tend to have a higher percentage of change after correction than men.

## Discussion

We aimed to study mortality trends in three Latin America countries between 1985 and 2012 and we found that disparities remain: age-adjusted CHD mortality rates declined in Argentina and Colombia (51.1 and 6.5% respectively) and increased in Mexico (60.8%). Trends in young adults (< 35 years old) had a significant increment in the last decade in Mexico for men and women, but not in Argentina or Colombia. Garbage-code corrections produced dramatic increases in mortality rates in Argentina, particularly in women and amongst the young adults and the elderly.

Our results in changes in CHD mortality trends are consistent with those previously reported [7, 16], although some important differences in magnitude could be explained by the study period and age-groups considered. Pagan, et.al., studied mortality rates from 2000 to 2012 for adults 36 to 64 years old and had a similar behavior to what we observed in the three countries in the same period [7]. In Argentina, they observed a smaller reduction (19% men, 15% women) than ours (25% men, 24% women). In Colombia, the magnitude of the change was very similar, around zero for men in both studies and a 5% reduction in women (6% in our study). There are differences in both magnitude and direction in Mexico in this period. They reported an increase of 6% in men, which is lower than the 15% we found. In women, they reported a decrease of 6% and we found an increase of 5% [7]. These differences could be explained by the behavior of mortality in the younger and older groups, which were not included in their study.

The epidemic pattern experienced by Argentina is comparable to that reported in USA, Canada, France, Uruguay, Panama and the United Kingdom who experienced CHD mortality increases up to 1970, to be followed by a steady decrease since then [4]. The decreasing magnitude is similar to that in France and Estonia with a total percentage change between 46 and 50 in men and 53–55 in women [4]; in contrast, Uruguay and Panama also had decreases, but the magnitude was around 30%. Mortality rates in Colombia are also decreasing but at a smaller pace, percentage change on the overall period was 13.2% in men and 21.7% in women. In magnitude, these mortality rate reductions are comparable to those observed in Bulgaria, where total percentage change was 14.3 and 21.5 for men and women, respectively [4]. In the case of Latin American countries, Venezuela had similar changes to Colombia for mortality in women [7]. In contrast, CHD mortality rates in Mexico are still rising. These epidemic patterns are similar to those reported in the Russian Federation, Ukraine, and Latvia before 2002, although the magnitude of the increase in CHD mortality rates in these countries was higher [14]. In El Salvador, Sri Lanka and the Philippines both, epidemic form and magnitude changes, were similar to those in Mexico [7, 29].

Age and sex specific trends in Colombia, Mexico and Argentina are complex, but trends in young adults in Argentina and Mexico are worrisome. Very young Mexican adults (< 35 years) showed an increase in CHD mortality in the last decades. Mexican men 35–44 also had an increase in the study period, but the increase rate was lower. Stagnation of trends was observed only in very young Argentinian men, but it was not observed in the group of adults aged 35–44. Finally, in the Colombian population, CHD mortality rates tended to decrease in both age groups. Few studies have analyzed changes in the mortality trends in young adults and only some of them have found this increase or stagnation [3, 7, 10, 11]. A study conducted in 26 European countries concluded that in most countries the decline rate observed in younger age groups is similar to that observed in older populations [14]. However, in a small number of countries, like the United Kingdom or the United States, the population under 55 years have experienced smaller decreases in CHD mortality rates since 1990 than those observed in previous years and older age groups [3, 10]. In the analysis performed by Pagan, et.al., Latin America rate increases in men were observed in Costa Rica, Mexico, and Panama, while for women they were only observed in Mexico and Panama [7]. This analysis was restricted to the 35 to 44 years group, while we observed declines in the Mexican population and stagnation in Argentinian men aged 35 years and under.

Several reports have warned against the crude comparison of CHD mortality rates across countries without considering the quality of death certificates. All previous studies comparing rates across Latin America have reached the conclusion that Argentina is experiencing one of the lowest CHD mortality rates. However, after garbage-code correction, we observed that Argentina was not a low risk country at the beginning of the study period, with rates comparable to Mexico or Colombia. Garbage-code correction in these countries did not affect slopes, suggesting that the quality of reporting for CHD has not changed between 1997 and 2015. Presumably, garbage codes are more commonly used in the youngest and oldest members of the population because they are unspecific. It is difficult to diagnose coronary heart disease in very young adults because it is a chronic disease and it is uncommon to develop it at younger ages. On the other hand, elderly people might have more than one underlying disease and unspecific codes simplify the selection of the underlying cause of death. It is important to mention that while the method for garbage-code correction used in our paper has shown to be consistent throughout multiple studies [27, 28], no method has been established as the “gold standard” and errors in the comparison across countries could still remain.

Previous studies suggest that only a few countries have good-quality on CHD mortality statistics, requiring correction methods to adjust for CHD registry limitations [25, 28]. In our study, we confirmed that the garbage-code correction is fundamental to improve the comparison of CHD mortality patterns. In our case, the correction particularly affected Argentina, where a high proportion of records were coded with unspecific mortality codes; yet, in countries with better quality of data, such as Colombia and Mexico, the correction produced smaller changes. We strongly recommend the use of the correction method while analyzing CHD mortality trends, unless there is certainty about the reliability of CHD mortality coding. Countries need to promote the improvement of CHD mortality registration, highlighting the importance of accurate registry for policy decision making and evaluation [25, 27].

The three countries included in this analysis are upper-middle income countries with similar life expectancy at birth and demographic distribution [17–19]. Furthermore, the three of them experienced the epidemiological transition in the last decades of the twentieth century [30, 31]. However, there are some important differences: at the beginning of the study period CHD was the main cause of death in the three countries and, at the end of the period, diabetes was the main cause of dead in Mexico [18].

To our knowledge, no study on the contribution of risk factors to changes in CHD mortality in Latin America has been conducted. One study in Argentina analyzed the contribution of changes in risk factors and treatments in CHD mortality trends [32]. In this study, the authors found that evidence-based therapies accounted for approximately 49% of the deaths prevented or postponed; in contrast, changes in risk factors trends accounted only for 32.6% [32]. Although the main changes are due to medical treatments, especially hypertension treatments and secondary prevention after AMI, the reduction of three key risk factors could help to explain changes in mortality in Argentina: an important fall in hypertension prevalence (34.6%) [32], a 7% decrease in the smoking prevalence between 1980 and 2012 (from 26.6 to 19.8%) [33], and reductions in total cholesterol, which declined from 5.4 mmol/L in 1980 to 5.0 mmol/L in 2008 [34]. In contrast, modeling studies from other LMIC suggest that the main drivers of CHD mortality are preventable risk factors, such as diet and physical activity, and improvements in medical and surgical treatments [5, 12]. In our study, observed changes in Mexico and Colombia could be similarly explained by changes in risk factors. For instance, the reduction of two key two risk factors could explain changes in mortality rates in Colombia, where smoking decreased from 16.1% in 1980 to 11.2% in 2012, and total cholesterol in men decreased 1 mmol/L between 1980 and 2008 [33, 34]. In contrast, in Mexico, diabetes, BMI and total cholesterol did not have favorable changes. The prevalence of diabetes increased 68% from 1980 to 2014 (6.5 to 10.9) [35]. BMI increased in men from 25.5 kg/m^2^ in 1980 to 27.4 kg/m^2^ in 2008, and in women from 23.4 kg/m^2^ to 28.7 kg/m [36]. The contribution of these potential explanations needs to be further studied, taking into consideration the long latency of the disease.

Some important limitations must be mentioned. Like any other study that uses mortality data across multiple versions of the International Statistical Classification of Diseases (ICD), there is potential for attribution bias owing to both the change between versions of ICD and the procedures used to code deaths. Vital statistics come from an independent registry and are subject to errors, the use of garbage-code correction allows to diminish the effect of the quality of the information, but other mechanisms for bias could persist.

## Conclusions

Latin America is not a homogeneous region in terms of CHD mortality. CHD risk factor profiles of each country, and differences in health care access and quality could explain some of the observed differences across countries. While no study of the contribution of these factors to CHD mortality changes in Latin America has been conducted, modeling studies from other LMIC suggest that main drivers of CHD mortality are preventable risk factors, such as diet and physical activity, and the remaining part is attributable to improvements in medical and surgical treatments [5, 32]. Furthermore, the disease burden is even bigger after correcting for misclassification, which suggests the need of new strategies for improving CHD mortality data quality in the region. Although CHD mortality is falling in Argentina, the modest falls in Colombia and substantial rises in Mexico highlight the region’s urgent need for effective population-wide prevention policies.

## Data Availability

The dataset(s) supporting the conclusions of this article is(are) publicly available in the World Health Organization (WHO) database. http://apps.who.int/healthinfo/statistics/mortality/causeofdeath_query/start.php

## Appendix

**Table 1:** Age-standardized CHD mortality rates per 100,000 by country. 1985–2015

Sex	Argentina				Colombia				Mexico			
	Period	AMR^a^	Mortality rates (min-max)	APC^b^	Period	AMR^a^	Mortality rates (min-max)	APC^b^	Period	AMR^a^	Mortality rates (min-max)	APC^b^
ALL	1985–1988	136.3	133.4–137.8	−0.3	1985–2011	138.7	128.1–160.6	− 0.4^	1985–1989	89.3	83.3–99.3	6.0^
	1988–1994	117.8	103.8–135.2	−5.7^	2011–2015	141.1	128.3–150.7	4.1^	1989–1996	111.7	106.7–119.8	2.3^
	1994–1999	93.4	89.2–98.1	− 0.4					1996–2007	120.4	117.2–124.8	−0.3
	1999–2003	86.1	78.1–95.8	− 5.6^					2007–2015	128.8	115.4–140.8	2.1^
	2003–2009	69.5	62.2–75.1	−2.5^								
	2009–2015	64.6	62–67.3	0.6								
MALE	1985–1988	190.7	186.5–194.8	0.3	1985–2003	168.5	153.8–190.5	−0.7^	1985–1987	93.0	90.2–95.8	0.5
	1988–1994	169.3	153.3–191.3	−4.9^	2003–2006	162.2	156.4–168.8	2.5	1987–1990	104.6	94.8–111.9	9.3^
	1994–1999	138.0	132.5–142.5	−0.9	2006–2011	164.7	158.3–170.1	−1.1	1990–1996	130.4	125.1–137.9	2.1^
	1999–2008	115.9	102.4–142.1	−4.0^	2011–2015	175.6	157.6–186.9	4.7^	1996–2007	141.1	136.3–145.2	0.1
	2008–2015	94.3	91.7–98	0.2					2007–2015	157.0	139–173.1	2.5^
FEMALE	1985–1988	91.8	89.7–93.3	−0.4	1985–2012	113.5	103.5–134	2.5	1985–1989	81.8	76.7–91.9	6.6^
	1988–1994	77.5	66.1–91.2	−6.7^	2012–2015	115.4	104.8–122	−1.1	1989–1992	94.9	90.4–102.5	− 1.6
	1994–1999	59.1	56.2–62.6	0					1992–1996	97.6	92.3–102.7	3.5
	1999–2004	53.1	47–61.8	−6.4^					1996–2007	101.3	96.6–105.7	−0.8^
	2004–2015	42.2	38.9–44.9	−0.3					2007–2015	104.0	94.2–112.4	1.8^

Periods were identified by joinpoint regression analysis

^*p*-value < 0.01.

^a^*AMR* Average mortality rate

^b^*APC* Annual percentage change

**Table 2:** Age-standardized garbage- codes corrected CHD mortality rates per 100,000 by country. 1985–2015

Sex	Argentina				Colombia				Mexico			
	Period	AMR^a^	Mortality rates (min-max)	APC^b^	Period	AMR^a^	Mortality rates (min-max)	APC^b^	Period	AMR^a^	Mortality rates (min-max)	APC^b^
ALL	1997–1999	183.6	181–186.2	1.2	1997–2003	149.8	142.4–156.4	−1.2	1997–2007	135.0	128.9–142.4	−0.6^
	1999–2004	169.2	152.6–187.5	−5.0^	2003–2006	147.5	144.1–151.6	2.3	2007–2015	138.9	128.2–149.4	1.6^
	2004–2012	141.2	132.8–148	−0.6	2006–2011	147.4	140.3–153.3	−2.2				
	2012–2015	127.0	120.1–136.7	− 5	2011–2015	148.9	137.6–157.6	3.8^				
	APC^c^ = 104.6% *P*-value < 0.01~				APC^c^ = 9.4% *P*-value = 0.38~				APC^c^ = 10.2% *P*-value = 0.43~			
MALE	1997–1999	246.1	242.4–249.8	1.1	1997–2003	177.7	168.6–183.4	−1	1997–2007	154.8	150.8–160.6	−0.2
	1999–2004	227.5	207.7–251.7	−4.7^	2003–2006	177.4	171.5–183.9	2.5	2007–2015	167.0	151.4–182.3	2.1^
	2004–2012	187.1	178–196.1	−1.2	2006–2011	178.2	170.5–184.3	− 1.8				
	2012–2015	167.5	159.2–179.3	− 4.1	2011–2015	184.4	168.1–194.8	4.2^				
	APC^c^ = 83.3% *P*-value < 0.01~				APC^c^ = 8.5% *P*-value = 0.46~				APC^c^ = 8.2% *P*-value = 0.62~			
FEMALE	1997–1999	135.8	133.5–138.1	1.7	1997–2003	125.2	119.3–132.6	−1.5	1997–2007	116.7	108.4–125.2	−1.1^
	1999–2004	125.0	112.2–139.9	−5.3^	2003–2006	121.4	120.1–123.5	2.2	2007–2015	114.1	107.4–120.4	1.0^
	2004–2011	105.9	99.7–111.7	0.2	2006–2011	121.0	114.6–126.6	−2.5				
	2011–2015	98.4	89.9–110	−4.8^	2011–2015	120.0	111.9–128.1	3.7^				
	APC^c^ = 104.4 *P*-value = 0.02~				APC^c^ = 10.6 *P*-value =0.32~				APC^c^ = 12.8% *P*-value = 0.40~			

Periods were identified by joinpoint regression analysis.

^*p*-value < 0.01.

^a^*AMR* Average mortality rate.

^b^*APC* Annual percentage change

^c^Average percentage change between original and garbage-codes corrected mortality rates

## Abbreviations

AMI: Acute myocardial infarction
APC: Average Percentage Change
BIC: Bayesian Information Criterion
CHD: Coronary Heart Disease
GC: Garbage codes
ICD: International Classification of Diseases
LMIC: Low and middle income countries
